# A-FABP and oestrogens are independently involved in the development of breast cancer

**DOI:** 10.1080/21623945.2019.1690827

**Published:** 2019-11-22

**Authors:** Bing Li, Jiaqing Hao, Xiaofang Yan, Maiying Kong, Edward R. Sauter

**Affiliations:** a Department of Microbiology and Immunology, University of Louisville, Louisville, KY, USA; b Department of Bioinformatics and Biostatistics, University of Louisville, Louisville, KY, USA; c Division of Cancer Prevention, National Cancer Institute, 9609 Medical Center Drive, Rockville, MD, USA

**Keywords:** Breast cancer, A-FABP, oestrogens, obesity, menopause

## Abstract

We previously reported that postmenopausal obese women exhibit increased levels of circulating adipocyte fatty acid binding protein (A-FABP), which is associated with breast cancer (BC) development. In postmenopause, increased oestrogen levels are reported to be associated with increased BC risk. Herein, we assessed if oestrogens, including oestrone (E1), oestradiol (E2) and oestriol (E3), are associated with A-FABP in the obesity-related BC development. We collected 249 serum samples from women with or without BC and measured serum levels of E1, E2, E3 and A-FABP. Considering all subjects, E1 and E2 but not E3 levels were significantly higher in pre- than in postmenopause individuals. E3 and E1 levels were higher in non-obese than in obese women. When samples were separated by BC status, E2 levels were significantly higher, while E1 and E3 levels were significantly lower in postmenopausal obese than non-obese women without BC. These differences based on body mass index (BMI) were not observed among women with BC. E3 levels were higher in obese women with BC than those without. A-FABP levels were significantly higher in postmenopausal obese women regardless of BC status. In addition, A-FABP was not associated with E1, E2 or E3. Altogether, our data suggest that A-FABP is independently regulated by obesity and menopausal status compared to oestrogens, thus playing a unique role in the development of BC.

## Introduction

FABPs consist of a family of at least nine members of 14–15 kDa intracellular proteins which have been shown to play a central role in regulating inflammatory and metabolic pathways [1,2]. Different FABP members exhibit distinct tissue expression profiles. For example, A-FABP (also known as FABP4) is predominantly expressed in adipose tissue while epidermal FABP (E-FABP, FABP5) is the major FABP in the epidermis [3–5]. Our recent studies demonstrate that A-FABP expression in macrophages promotes pro-tumour functions of tumour associated macrophages (TAM) in mouse models of breast cancer (BC) [6,7]. Moreover, A-FABP is secreted by adipose tissue to promote obesity-associated BC [8]. Thus, A-FABP represents a newly identified factor contributing to BC development.

Oestrogens are known drivers of hormone sensitive BC, and hormone receptor status is a basis for treatment of BCs, most of which express the oestrogen receptor (ER) and/or progesterone receptor [9]. Obesity is a known risk factor for postmenopausal BC, and obesity increases risk of disease recurrence and all-cause mortality in BC survivors [10]. However, the relationship among these factors, including A-FABP, oestrogens, obesity and menopausal status, related to BC development remain largely unknown.

There are three oestrogens produced in the body, oestrone: E1, oestradiol: E2, and oestriol: E3. E2 is the most potent oestrogen [11], although all three have oestrogenic potential. The relative serum concentration of E1 and E2 varies with menopausal status [12]. The primary source of E1 and E2 in premenopausal women is the ovary, whereas in postmenopausal women the primary source is through aromatization (of adrenal androstenedione for E1, of adrenal testosterone for E2) [13,14]. The primary source for E3 in nonpregnant women is the irreversible conversion from E1 [12]. High levels of E3 are produced during pregnancy by the placenta. Aromatase production increases with increased body mass index (BMI) [15]. There have been multiple studies evaluating the association of E2, and to a lesser extent E1, with BC. A study evaluating postmenopausal BC survivors found that serum levels of E1 and E2 were significantly lower among overweight and obese survivors who had lost ≥ 5 pounds of body weight 6 months after starting the study compared to those who had not [10]. E3 is the most highly produced oestrogen in the body, though circulating levels of E3 are lower than E1 and E2 due to a higher rate of metabolism and excretion [16]. E3 is used to treat menopausal symptoms [17]. It has been found to reduce hot flashes, improve vaginal atrophy, and reverse the postmenopausal decline in skin thickness and collagen content. It can cause proliferation of breast epithelium when administered at sufficiently high doses [12]. Relatively little is known about the carcinogenic effects of E3. At one point it was thought that E3 might play a protective role in BC development [18], but findings from subsequent assessment of its role in BC development have been mixed [19]. Both E2 and E3 have been found to induce mammary tumours [20]. Given that the factors that drive BC development are likely to vary in different settings, such as obesity vs. leaner body weight, and pre- vs postmenopause, the associations of individual oestrogens in different settings deserve further investigation.

Since both oestrogens and A-FABP have been linked to obesity and to obesity related breast cancer, it is important to understand if they work together or through independent pathways. In the current study we collected serum from 249 female non-BC (healthy) and BC subjects, and assessed circulating levels of the three oestrogens and A-FABP. We analysed their possible associations with obesity, menopausal status and BC development.

## Methods

### Human samples

Serum samples from 249 patients with or without breast cancer were collected in double-blind fashion as described before [8]. All participants provided informed consent to an Institutional Review Board-approved protocol. The samples were classified into BC and non-cancer groups according to their clinical diagnosis, and into premenopause or postmenopause by their menstrual history. Based on BMI they were separated into non-obese (BMI<30) or obese (BMI≥30) groups.

### Immunoassays

#### E1

Oestrone levels were determined by a solid phase enzyme-linked immunosorbent assay (ELISA) based on the typical competition principle (DB52051, IBL International). The unknown amount of E1 in human serum samples competed with a fixed amount of enzyme labelled E1 for the well-coated antibodies. The levels of oestrone in human samples were inversely proportional to the intensity of the developed colour in the ELISA wells and calculated using the standard curve.

#### E2 and E3

The levels of E2 and E3 in human samples were measured by Oestradiol (Cat# 582,251) and Oestriol (Cat# 582,281) ELISA kits (Cayman Chemical), respectively. Similar to E1 measurement, these ELISA kits were used for quantification of E2 and E3 in human serum samples based on the competitive assay principle. Data were plotted as logit(B/B_0_) vs. log concentrations and sample concentrations were determined according to the B/B_0_ value of individual samples.

#### A-FABP

A-FABP levels in human samples were measured using an enzyme immunoassay kit (EIA) (Cat# A05181) from SPI Bio. A human A-FABP specific antibody coated in the plate captured A-FABP in the serum, which bound to biotin-labelled polyclonal anti-human A-FABP. This double-antibody sandwich immobilized streptavidin-horseradish peroxidase (HRP) and catalysed hydrogen peroxide/TMB solution to form a yellow compound. The intensity of the colour was proportional to the A-FABP levels in the human serum.

### Statistical analysis

Oestrogen levels were summarized by mean and standard deviation (SD) and sample size, stratified by obese (obese vs. non-obese) and menopause status (pre vs post) for each type of oestrogen. The comparison between two groups was carried out using the Manny-Whitney test (two sided), which is a rank based nonparametric test and is free of normal assumptions on the distribution of the data[21]. Multiple linear regression models [21] were used to examine the relationship between A-FABP and oestrogen level with control of obese and menopause status, which provides a more accurate approach to estimate the association between A-FABP and oestrogen level than their correlation coefficients. P values less than 0.05 were considered statistically significant.

## Results

Demographic and clinical characteristics of 249 patient samples are shown in Table 1. Clinical parameters including age, BMI, race and menopausal status were comparable between non-BC and BC groups. We did not observe any significant differences in overall levels of oestrogens and A-FABP between the two groups. As BC development has been associated with menopausal and obese status, we further analysed the association of each parameter with different patient subsets.

**Table 1.** Demographics and clinical characteristics of the patients.

Characteristics	Non-Cancer patients	Cancer patients
Number (n)	157	92
Age, years, mean (range)	48 (19–67)	53 (36–83)
BMI, kg/m^2^ (range)	27.6 (16.3–44.4)	28.5 (16.8–47.8)
Race	151W,3B, 2H, 1A	87W,4B,1A
Menopausal status (n)	Pre (63) Post (94)	Pre (39) Post (53)
Stage (n)	N/A	DCIS (17), I (29), II (35), III (11)
Oestrone (E1) (pg/ml) (mean±SD)	106 ± 199.2	133.9 ± 114.6
Oestradiol (E2) (pg/ml) (mean±SD)	77.4 ± 98.5	72.6 ± 68.2
Oestriol (E3) (pg/ml) (mean±SD)	40.7 ± 30.9	41.2 ± 37.3
A-FABP (ng/ml) (mean±SD)	25.32 ± 18.97	25.3 ± 21.48

W: White, B: Black, H: Hispanic, A, Asian.

### Analysis of E1 with menopause, obese and cancer status

We first evaluated the association of E1 with menopause and obese status in the entire population. Expression of E1 was significantly higher in premenopause, and was associated with lower body mass in both pre- and postmenopausal women (Table 2), consistent with the known production of E1 by the ovary and suggesting that obesity might inhibit E1 production. To assess the potential influence of E1 on BC, we separated the data based on BC status. Consistently, E1 levels were significantly higher in pre- than in postmenopausal women regardless of BC status. Obesity-associated E1 inhibition was observed in both non-BC and BC patients, but only healthy postmenopausal women reached the significant difference (p = 0.005) (Table 3), Moreover, E1 levels were significantly upregulated in BC patients that were premenopausal and non-obese (p = 0.001), and E1 levels trended higher in both pre- and postmenopausal obese women with BC (Table 4). Altogether, our analyses suggest that E1 may promote BC development in premenopausal women, in particular in the non-obese group.

**Table 2.** Levels of oestrogens (E1, E2, E3) (pg/mL) in the entire population.

		Premenopause	Postmenopause	P value
	**Non-obese**	177.8 ± 225.6 (73)	99.0 ± 173.2 (102)	**< 0.001**
E1	**Obese**	101.7 ± 85.1 (29)	65.2 ± 48.0 (45)	**< 0.001**
	**P value**	**0.011**	**0.024**	
	**Non-obese**	102.4 ± 106.5 (73)	61.2 ± 91.8 (102)	**< 0.001**
E2	**Obese**	83.53 ± 8.9 (29)	59.7 ± 56.3 (45)	**< 0.001**
	**P value**	0.96	0.075	
	**Non-obese**	46.0 ± 33.1 (73)	46.7 ± 38.6 (102)	0.57
E3	**Obese**	25.6 ± 15.1 (29)	29.4 ± 22.3 (45)	0.47
	**P value**	**<0.001**	**0.004**

**Table 4.** Levels of oestrogens (E1,E2,E3) (pg/mL) based on obese status.

		Non-obese			Obese		
		Non-cancer	Cancer	P value	Non-cancer	Cancer	P value
E1	**Premenopause**	150.2 ± 267.6 (43)	217.4 ± 140.9 (30)	**0.001**	82.9 ± 53.8 (20)	143.4 ± 124.9 (9)	p > 0.05
	**Postmenopause**	101.7 ± 203.8 (71)	93 ± 63.9 (31)	p > 0.05	57.1 ± 55.2 (23)	73.7 ± 38.4 (22)	p > 0.05
E2	**Premenopause**	99.2 ± 121.2 (43)	107 ± 82.8 (30)	p > 0.05	88.9 ± 35.4 (20)	71.6 ± 45.5 (9)	p > 0.05
	**Postmenopause**	63.3 ± 102.8 (71)	56.5 ± 60.7 (31)	p > 0.05	70.1 ± 65.1 (23)	48.8 ± 44.4 (22)	p > 0.05
E3	**Premenopause**	47.1 ± 25.4 (43)	44.4 ± 42.2 (30)	p > 0.05	19.2 ± 10.2 (20)	39.8 ± 14.7 (9)	**0.001**
	**Postmenopause**	49.4 ± 35.8 (71)	40.3 ± 44.4 (31)	p > 0.05	20.8 ± 14.3 (23)	38.5 ± 25.6 (22)	**0.003**

**Table 3.** Levels of oestrogens (E1, E2, E3) (pg/mL) based on breast cancer status and according to pre/post-menopausal groups.

		Non-Cancer			Cancer		
		Premenopause	Postmenopause	P value	Premenopause	Postmenopause	P value
	**Non-obese**	150.2 ± 267.6 (43)	101.7 ± 203.8 (71)	**<0.001**	217.4 ± 140.9 (30)	93 ± 63.9 (31)	**< 0.001**
E1	**Obese**	82.9 ± 53.8 (20)	57.1 ± 55.2 (23)	**0.002**	143.4 ± 124.9 (9)	73.7 ± 38.4 (22)	**< 0.001**
	**P value**	0.2	**0.005**		0.11	0.31	
	**Non-obese**	99.2 ± 121.2 (43)	63.3 ± 102.8 (71)	**< 0.001**	107 ± 82.8 (30)	56.5 ± 60.7 (31)	**< 0.001**
E2	**Obese**	88.9 ± 35.4 (20)	70.1 ± 65.1 (23)	**< 0.001**	71.6 ± 45.5 (9)	48.8 ± 44.4 (22)	**< 0.001**
	**P value**	0.31	**0.035**		0.24	0.66	
	**Non-obese**	47.1 ± 25.4 (43)	49.4 ± 35.8 (71)	0.9	44.4 ± 42.2 (30)	40.3 ± 44.4 (31)	0.57
E3	**Obese**	19.2 ± 10.2 (20)	20.8 ± 14.3 (23)	0.73	39.8 ± 14.7 (9)	38.5 ± 25.6 (22)	0.47
	**P value**	**<0.001**	**<0.001**		0.58	0.44	

### Analysis of E2 with menopause, obese and cancer status

We then evaluated the association of E2 with menopause and obese status. Expression of E2 was significantly higher in pre- than in postmenopausal women, regardless of body mass (Table 2), consistent with the known production of E2 by the ovary. This observation was true both for women with and without BC (Table 3). Whereas E1 levels were significantly lower in obese than non-obese healthy postmenopausal women, the opposite was true for E2, where levels were significantly higher (Table 3). BC status did not significantly influence E2 levels (Table 4).

### Analysis of E3 with menopause, obese and cancer status

Compared to E1 and E2, E3 levels were lower and similar among pre- and postmenopausal women, suggesting that ovarian function is not critical for E3 production (Table 2). Interestingly, E3 levels were significantly higher in non-obese than in obese women, regardless of menopausal status (Table 2), suggesting that obesity significantly inhibits E3 production. When we compared E3 levels based on cancer status, we observed lower E3 expression in obese women without BC, but not among those with BC (Table 3). E3 expression was significantly higher among obese women with BC, regardless of menopausal status (Table 4).

### A-FABP is significantly increased only in obese postmenopausal women

We previously observed that A-FABP levels are higher in obese but not lean women with BC, and that A-FABP levels were higher overall in post- than premenopausal women[8]. Given the importance of menopausal status in BC risk and therapy, we evaluated A-FABP levels in cancer and non-cancer patients who also underwent E1, E2, and E3 assessment. A-FABP levels were significantly higher in obese postmenopausal with and without BC (Table 5), but not in premenopausal women, regardless of BC status, although in women with BC the difference approached significance (p = 0.053).

**Table 5.** A-FABP levels (ng/ml) based on breast cancer, menopause and obese status.

	Non-Cancer			Cancer		
	Premenopause	Postmenopause	P value	Premenopause	Postmenopause	P value
Non-obese	18.9 ± 17.6(43)	26.9 ± 16.2(71)	**0.008**	12.1 ± 14.6 (30)	24.9 ± 18.0 (31)	**0.005**
Obese	16.8 ± 16.7(20)	40 ± 22.5(23)	**0.001**	23.2 ± 16.1 (9)	44.8 ± 22.2 (22)	**0.012**
P value	0.642	**0.012**		0.053	**0.002**	

### Association of A-FABP with individual oestrogen members

**Figure 1.**
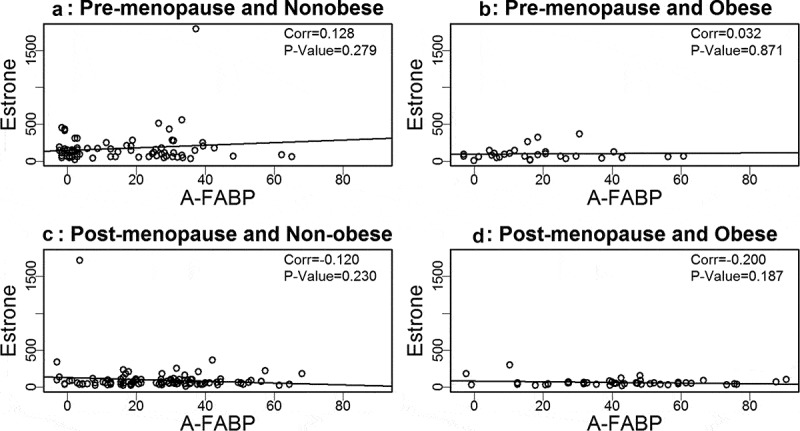
Analysis of the association between estrone and A-FABP.

**Figure 2.**
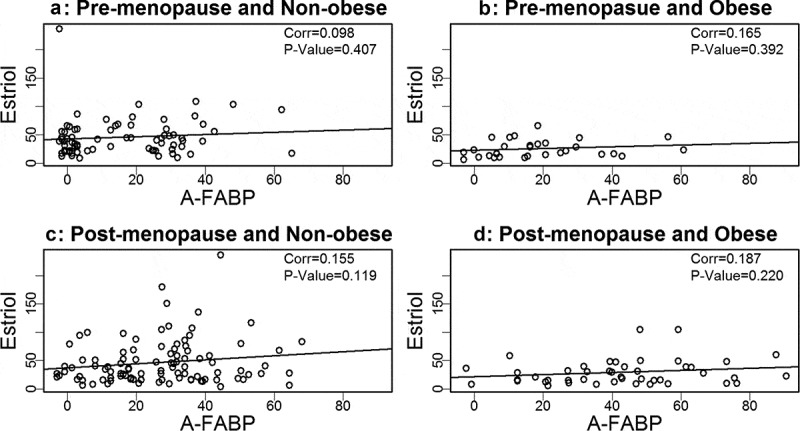
Analysis of the association between estriol and A-FABP.

**Figure 3.**
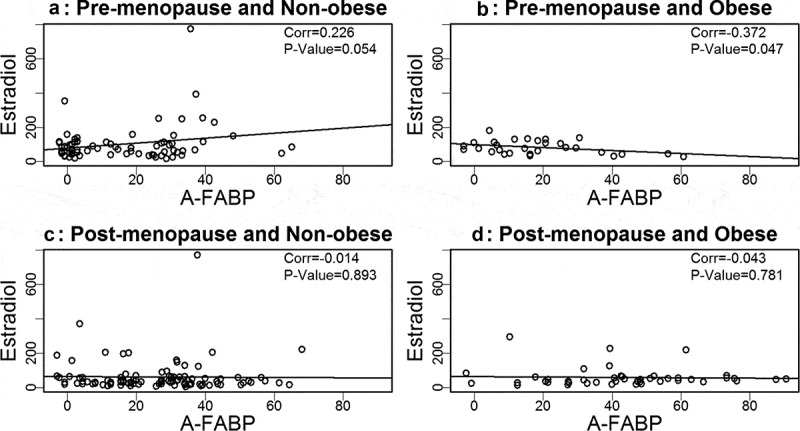
Analysis of the association between estradiol and A-FABP.

Based on our prior findings that A-FABP levels are elevated in obese and postmenopausal women, and elevated A-FABP promotes obesity-associated BC development[8], we wondered if one or more oestrogens would be associated with A-FABP. To address this question we developed linear regression models to examine the relationship between A-FABP and each oestrogen. When the data were analysed considering obese, cancer and menopause status, we did not find a significant correlation between A-FABP and E1, E2 or E3 (Figures 1–3).

## Discussion

There are three major endogenous oestrogens in humans, E1, E2 and E3. The primary driver of E1 and E2 synthesis in premenopausal women is the ovary, whereas in postmenopausal women E1 and E2 are predominantly produced in adipose tissue [9]. On one hand, oestrogen is strongly linked with a variety of cancers, including BC [9]. On the other hand, high dose oestrogen is used as a treatment for advanced postmenopausal BC [22]. As the most potent of the three oestrogens, E2 has received the most research focus. Oestrogens bind to ERα and ERβ, which in turn influence the expression of many genes[9]. Whereas E1 and E2 have high binding affinity for ERα, the binding affinity of E3 has been reported to be higher for ERβ [12]. Moreover, the dissociation of E3 from the ER is much faster than for E2, decreasing its potential (compared to E2) to induce cell proliferation [12]. E3 has potentially favourable immunomodulatory effects that are distinct from E1 and E2 [23]. At recommended doses for the treatment of menopausal symptoms E3 is not stimulatory to the uterus, whereas at higher doses it is [12].

A recent evaluation of various hormone replacement therapy (HRT) regimens demonstrated that, compared to no history of HRT use, women currently using oestrogen-only HRT were at slightly increased BC risk (odds ratio: OR = 1.08), oestrogen plus progestogen use risk was (OR = 1.77), relative risk among those taking E2 was 1.12, relative risk among those taking conjugated oestrogens (e.g., Premarin ®) was 4.47, whereas the relative risk among those taking E3 was 0.76 [24]. These findings suggest that at relatively low concentrations E3 may compete with E1 and E2 for ER binding, thereby having a receptor blocking function.

The Study of Women’s Health Across the Nation (SWAN) enrolled women 42–52 years old, BMI 22–30 kg/m [2], who had not undergone surgical removal of the uterus and/or both ovaries, were not on exogenous hormone medications, and had active menstrual function. Blood was collected for a period spanning 4 years before to 4 years after the final menstrual period (FMP). Serum was analysed for E1 and E2 [14]. Comparing samples collected 4 years prior to samples collected 4 years after the FMP [14], there was a 22% decrease in E1, a 69% decrease in E2 and a 32% decrease in androstenedione, the precursor to E1. The findings support the important contribution of adrenal androgens (including androstenedione) to postmenopausal oestrogen levels.

We observed that E1 and E2, but not E3, levels were significantly higher in pre- than postmenopausal women, consistent with the known ovarian production of E1 and E2 (but not E3) during premenopause. By contrast, E3 expression was similar regardless of menopausal status. Our observations of significantly lower levels of E1 and E3 in obese vs. non-obese premenopausal women (Table 2) are consistent with the report by the World Cancer Research Fund/American Institute for Cancer Research, which concluded that there was strong evidence that obesity decreases BC risk in premenopausal women, whereas being obese throughout adulthood, or gaining weight during adulthood, increases the risk of postmenopausal BC [25].

E1 levels trended higher in pre- and postmenopausal obese BC patients, and E3 levels were significantly higher in obese pre- and postmenopausal BC patients (Table 4). These findings are consistent with the association of oestrogen levels with obesity related postmenopausal BC [10]. While it was previously reported that oestrogens, including E3, are elevated in postmenopausal breast cancer, our data suggest that for E3 this difference is limited to obese women [26]. Whereas E3 levels in obese healthy women were on average less than half those of non-obese women, E3 levels in obese women with BC were similar to those in non-obese women and significantly higher than in obese women without BC, regardless of menopausal status. E3 levels are highest in pregnant women, a time of high oestrogen concentration. A recent report identified multiple genes involved in the development of cancer that are differentially expressed by the mammary gland in response to E3. The authors speculated that the altered gene expression after exposure to E3 may predispose the gland to cancer development, including the increased short term risk of BC that is present after pregnancy [27]. While chance cannot be excluded, the lower values among obese healthy vs. obese BC patients are consistent with higher concentrations of E3 driving obesity-associated BC.

We previously reported that circulating A-FABP, which is mainly secreted by adipocytes in obese individuals, induces mammary tumour formation [8]. We and others have reported that A-FABP levels are increased in obese healthy individuals and in obese BC patients [8]. What was not previously reported, and very interesting, is that A-FABP is significantly upregulated in post- but not premenopausal women, both those with and without BC (Table 5). We did not find a significant association between A-FABP and E1, E2 or E3, when grouping subjects based on obesity or BC status. This suggests that A-FABP and oestrogen(s) are independent drivers of obesity related postmenopausal BC. The possible independent ability of these biologic markers to predict which women will develop BC deserves additional studies. Of note, we did not observe significant correlations of above markers with BC stages (data not shown).

In summary, we found that circulating levels of E1 and E2, but not E3, were significantly higher in pre- than postmenopausal women. E3 demonstrated the strongest negative association of the three endogenous oestrogens with body mass in healthy women, but E3 levels in obese women with BC were significantly higher than in those without, suggesting that BC counterbalances E3 levels in obese patients. Given that A-FABP is upregulated in obese postmenopausal women, our findings suggest that A-FABP and oestrogens play independent roles in the development of obesity related BC. Of note, our findings are subject to certain limitations. Although evidence suggests that local oestrogen levels in breast tissue rapidly equilibrate with plasma levels [28], circulating levels of oestrogens do not fully account for local oestrogen production, which is important in BC development.
